# Supplementary figures and images for: Senescent cells inhibit mouse myoblast differentiation via the SASP-lipid 15d-PGJ2 mediated modification and control of HRas
Source: eLife. 2024 Aug 28;13:RP95229. doi: 10.7554/eLife.95229 (PMC11357351; doi:10.7554/eLife.95229)

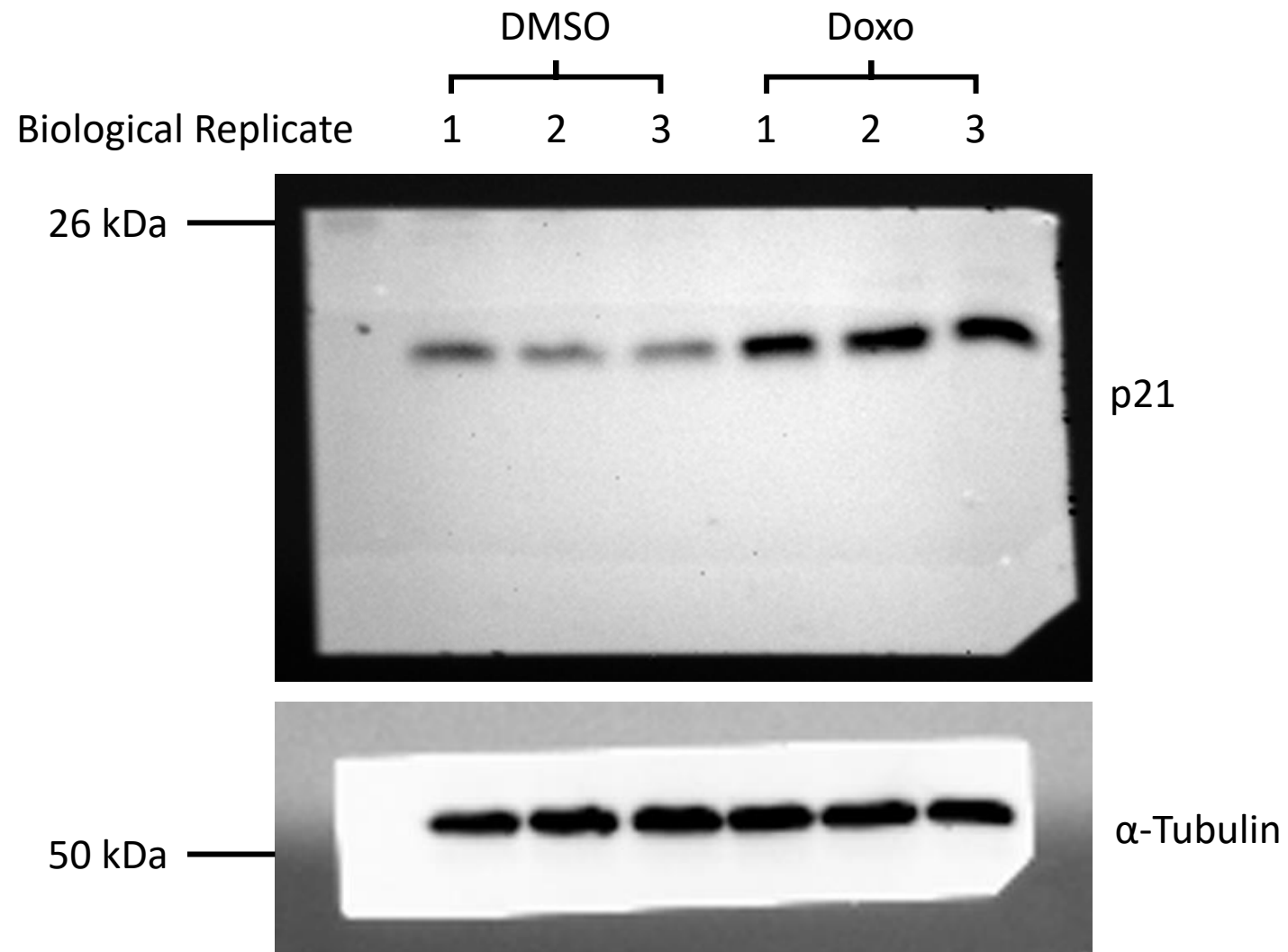

Supplement: Figure 1—source data 1. [file elife-95229-fig1-data1.zip › Figure 1-source data 1. Uncropped and labelled gels for Figure 1/Figure 1-source data 1. Uncropped and labelled gels for Figure 1.pdf]

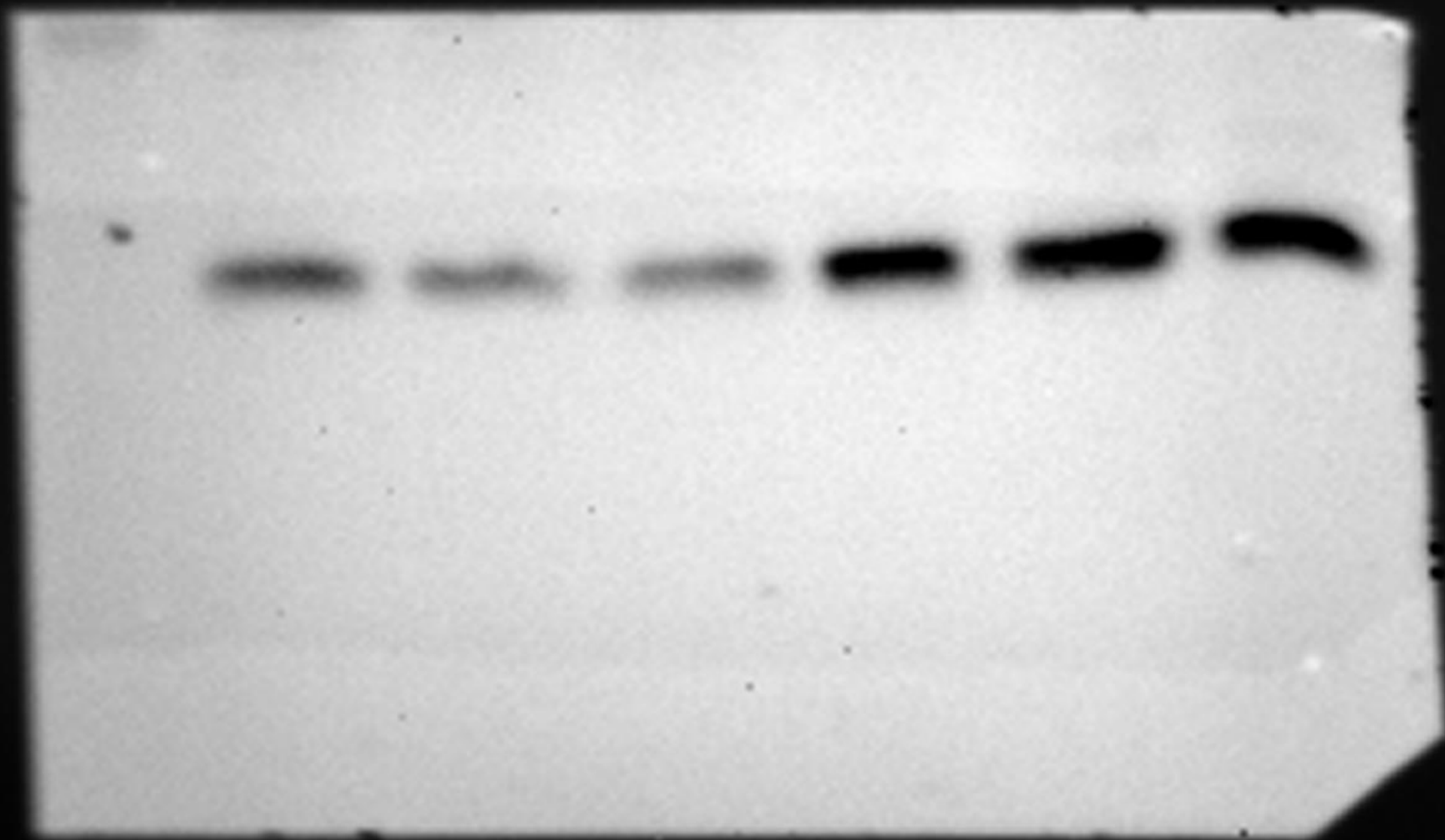

Supplement: Figure 1—source data 2. [file elife-95229-fig1-data2.zip › Figure 1-source data 2. Raw unedited gels for figure 1/Figure 1-source data 1. Raw unedited gels for figure 1.pdf]

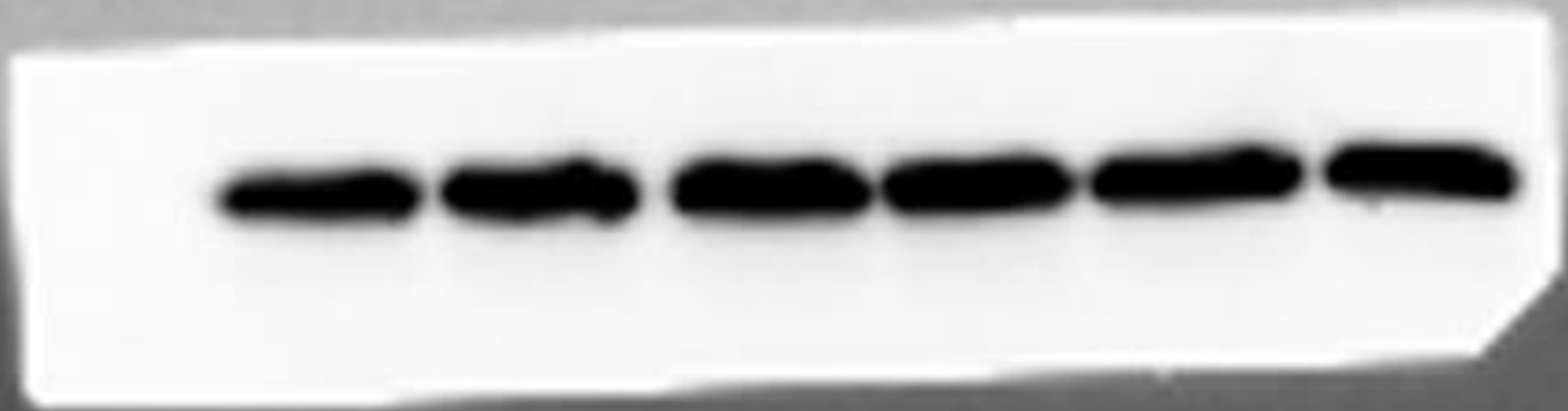

Supplement: Figure 1—source data 2. [file elife-95229-fig1-data2.zip › Figure 1-source data 2. Raw unedited gels for figure 1/Figure 1-source data 2. Raw unedited gels for figure 1.pdf]

15d-PGJ<sub>2</sub> (μM)

0 1 2 4

315 kDa

238 kDa

MHC

70 kDa

42 kDa

α-Tubulin

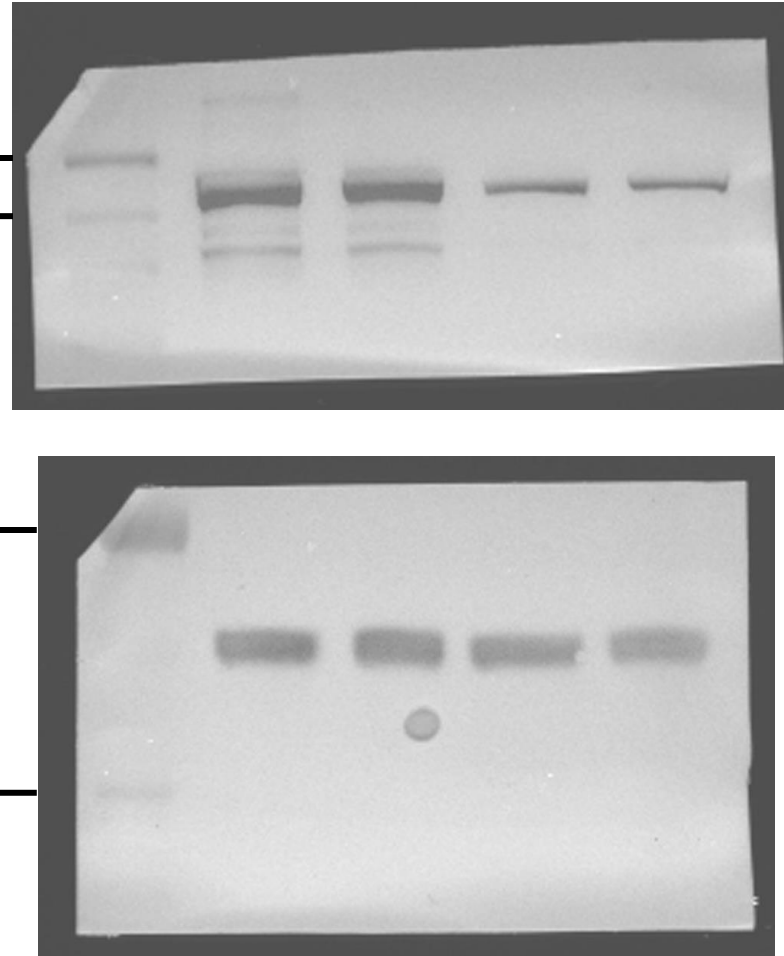

Supplement: Figure 2—source data 1. [file elife-95229-fig2-data1.zip › Figure 2-source data 1. Uncropped and labelled gels for Figure 2/Figure 2-source data 1. Uncropped and labelled gels for Figure 2.pdf]

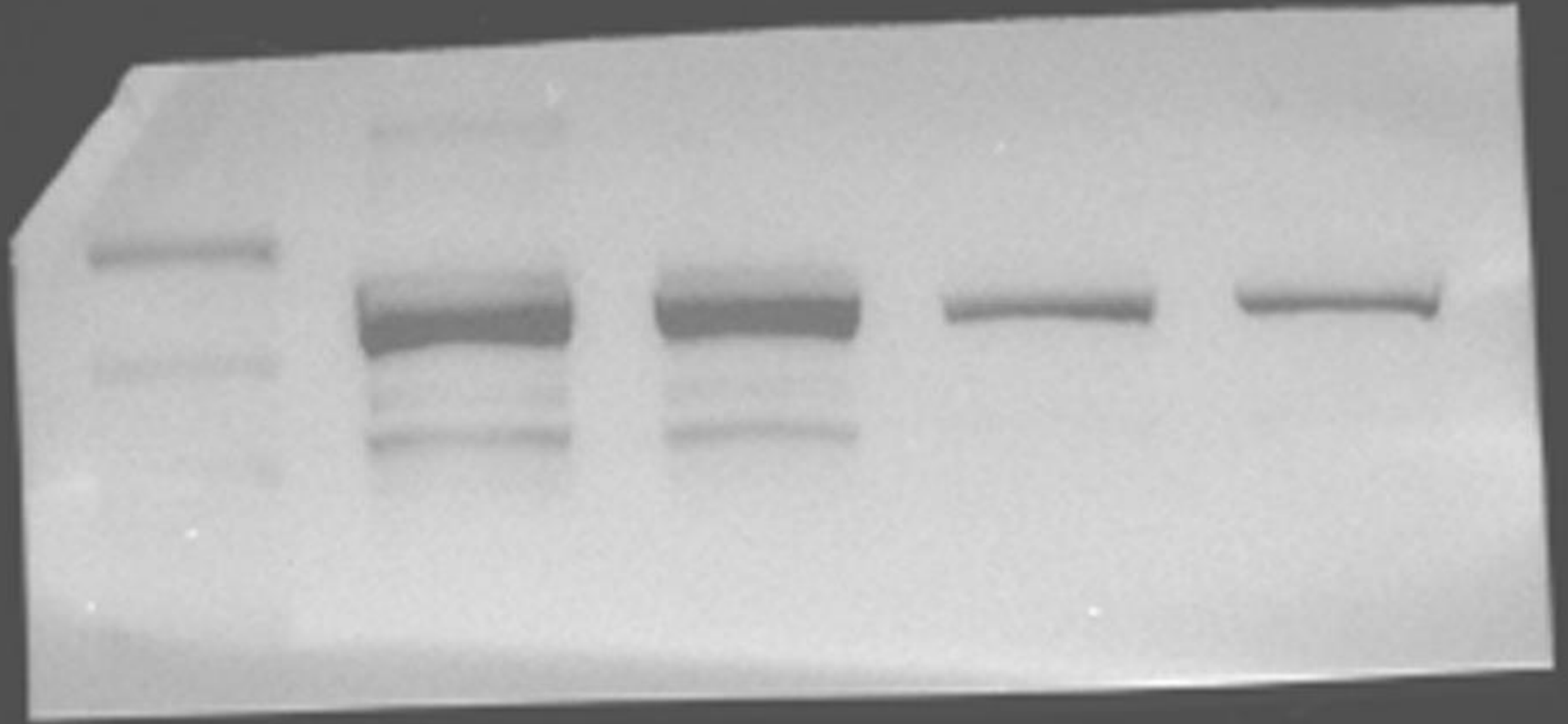

Supplement: Figure 2—source data 2. [file elife-95229-fig2-data2.zip › Figure 2-source data 2. Raw unedited gels for Figure 2/Figure 2-source data 1. Raw unedited gels for Figure 2.pdf]

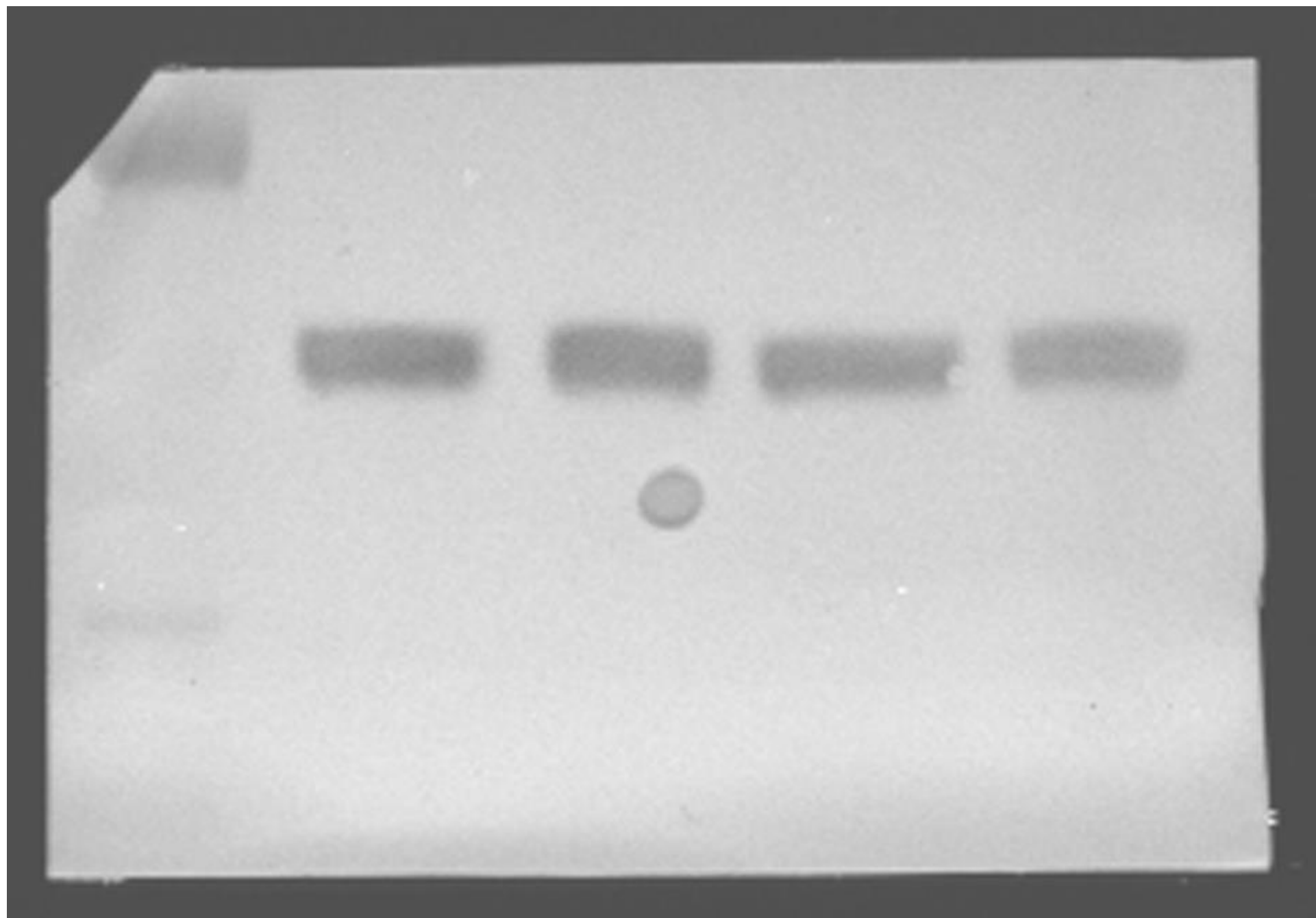

Supplement: Figure 2—source data 2. [file elife-95229-fig2-data2.zip › Figure 2-source data 2. Raw unedited gels for Figure 2/Figure 2-source data 2. Raw unedited gels for Figure 2.pdf]

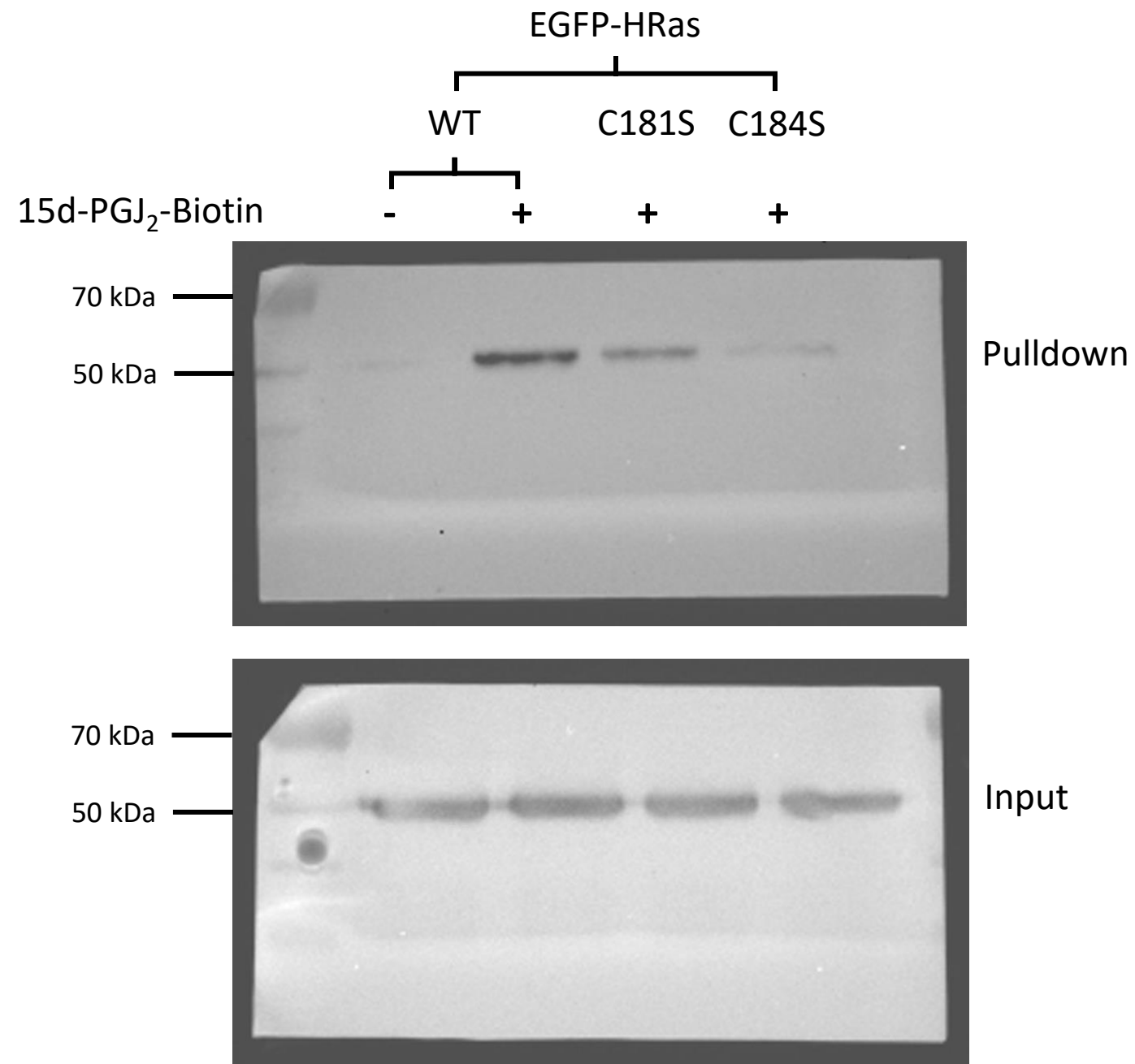

Supplement: Figure 3—source data 1. [file elife-95229-fig3-data1.zip › Figure 3-source data 1. Uncropped and labelled gels for Figure 3/Figure 3-source data 1. Uncropped and labelled gels for Figure 3.pdf]

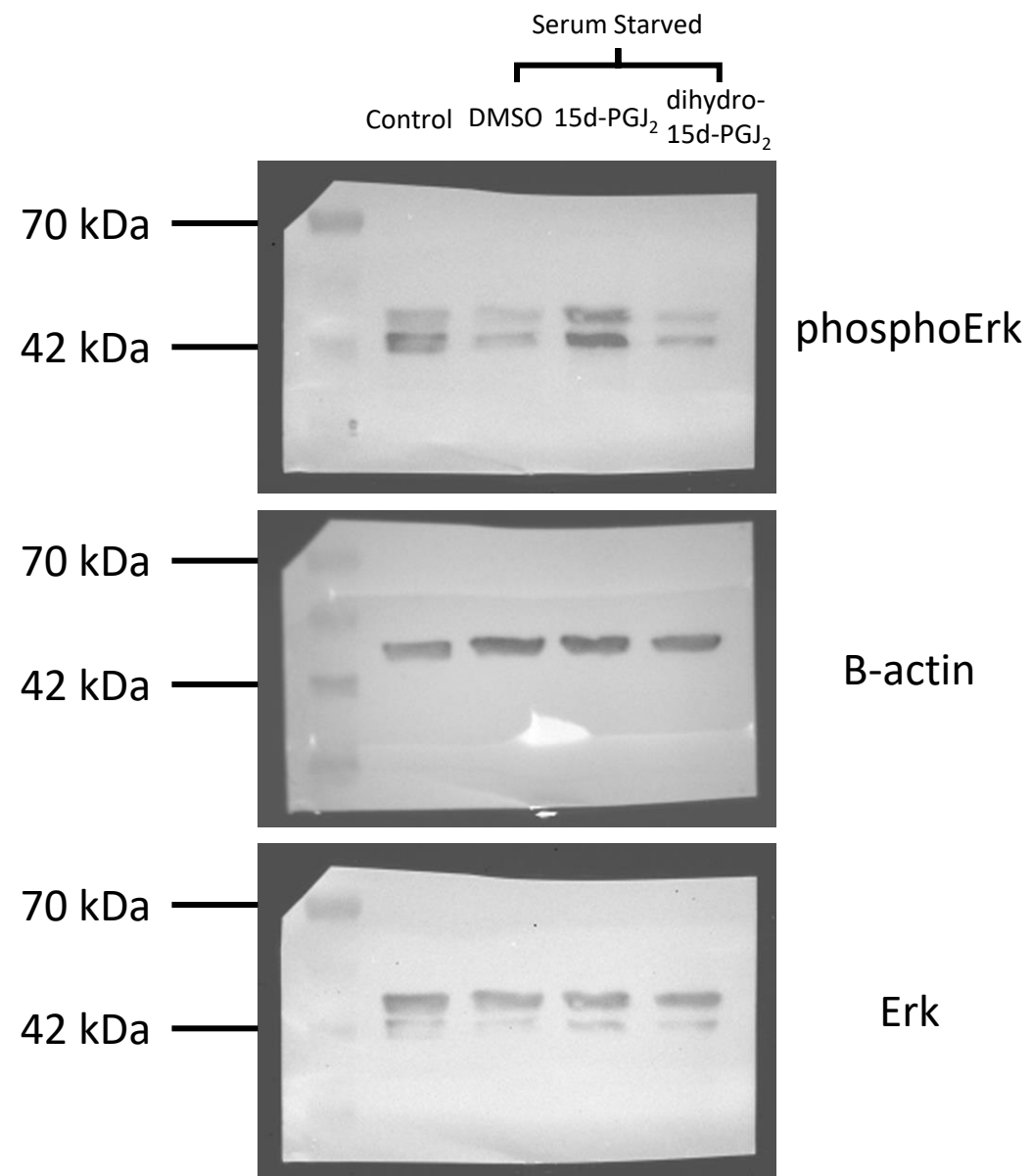

Supplement: Figure 3—source data 1. [file elife-95229-fig3-data1.zip › Figure 3-source data 1. Uncropped and labelled gels for Figure 3/Figure 3-source data 3. Uncropped and labelled gels for Figure 3.pdf]

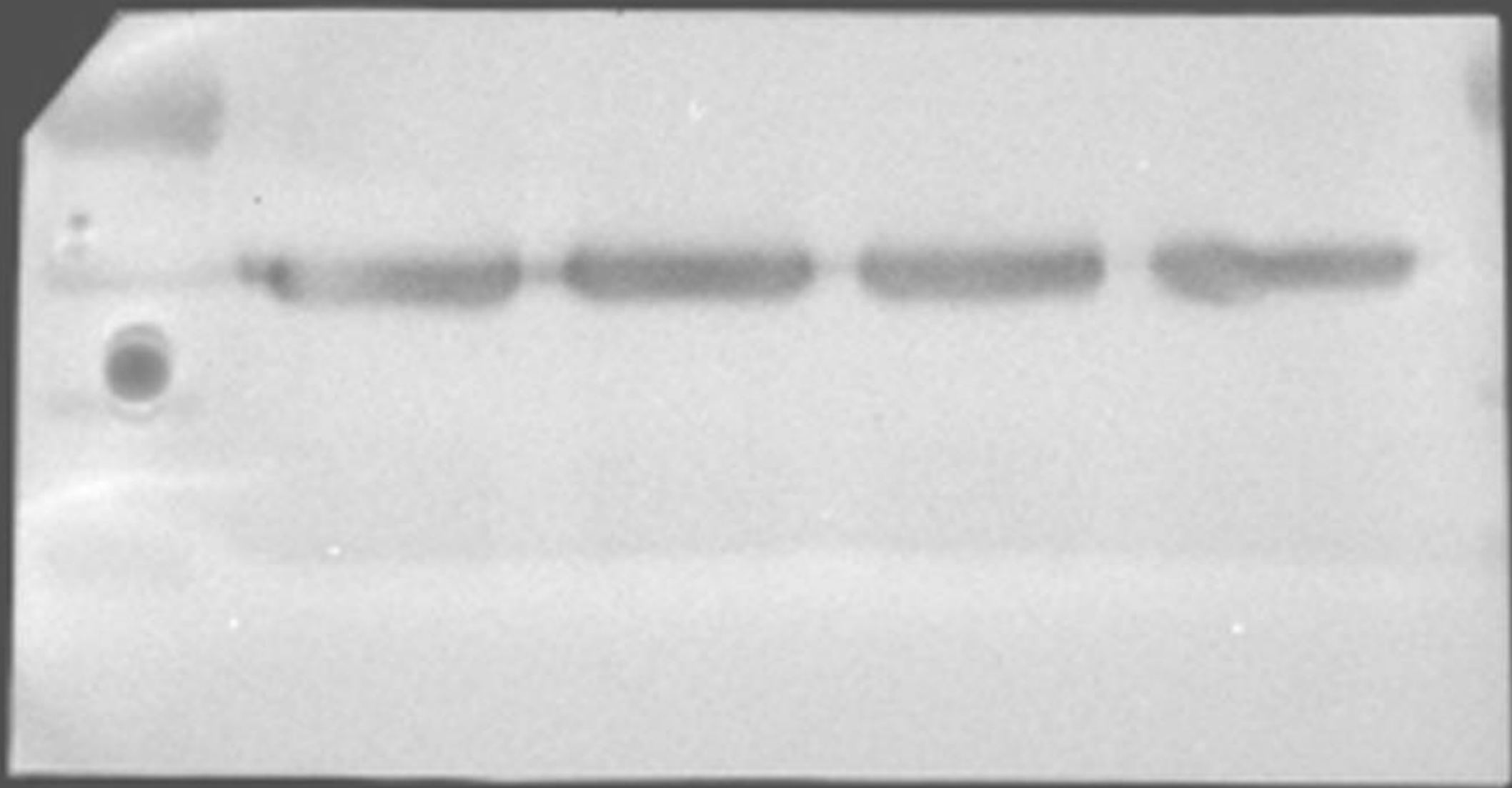

Supplement: Figure 3—source data 2. [file elife-95229-fig3-data2.zip › Figure 3-source data 2. Raw unedited gels for Figure 3/Figure 3-source data 1. Raw unedited gels for Figure 3.pdf]

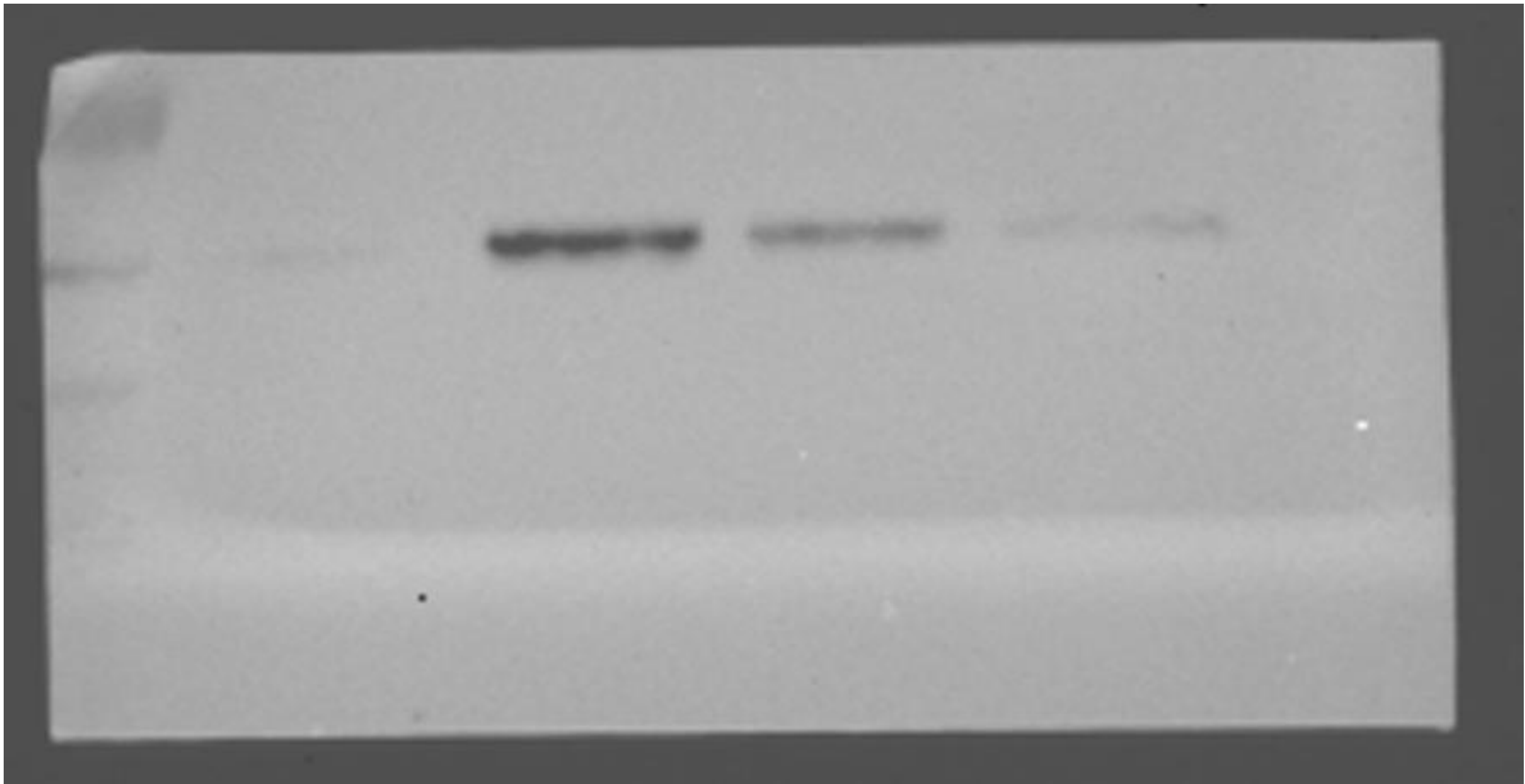

Supplement: Figure 3—source data 2. [file elife-95229-fig3-data2.zip › Figure 3-source data 2. Raw unedited gels for Figure 3/Figure 3-source data 2. Raw unedited gels for Figure 3.pdf]

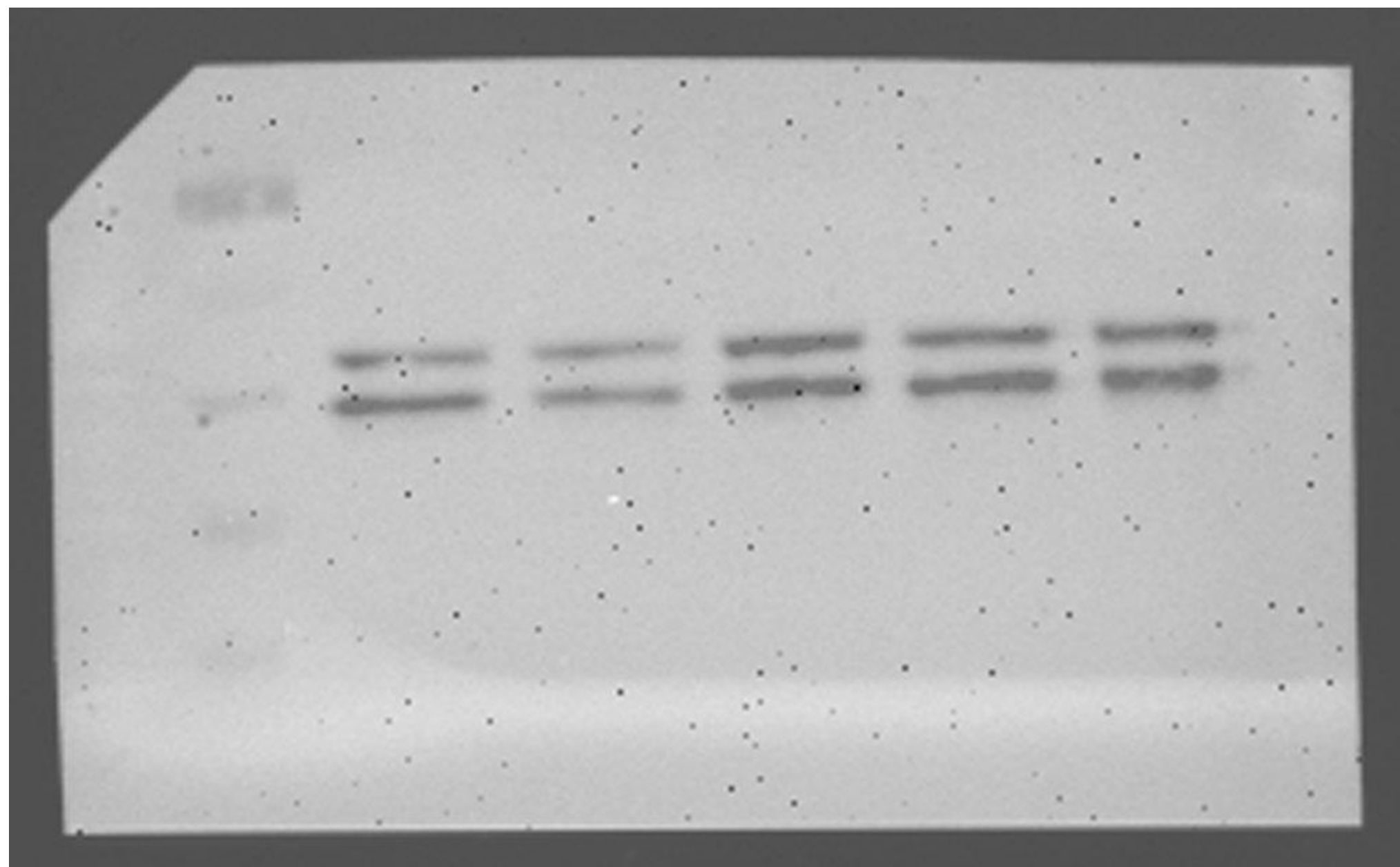

Supplement: Figure 3—source data 2. [file elife-95229-fig3-data2.zip › Figure 3-source data 2. Raw unedited gels for Figure 3/Figure 3-source data 3. Raw unedited gels for Figure 3.pdf]

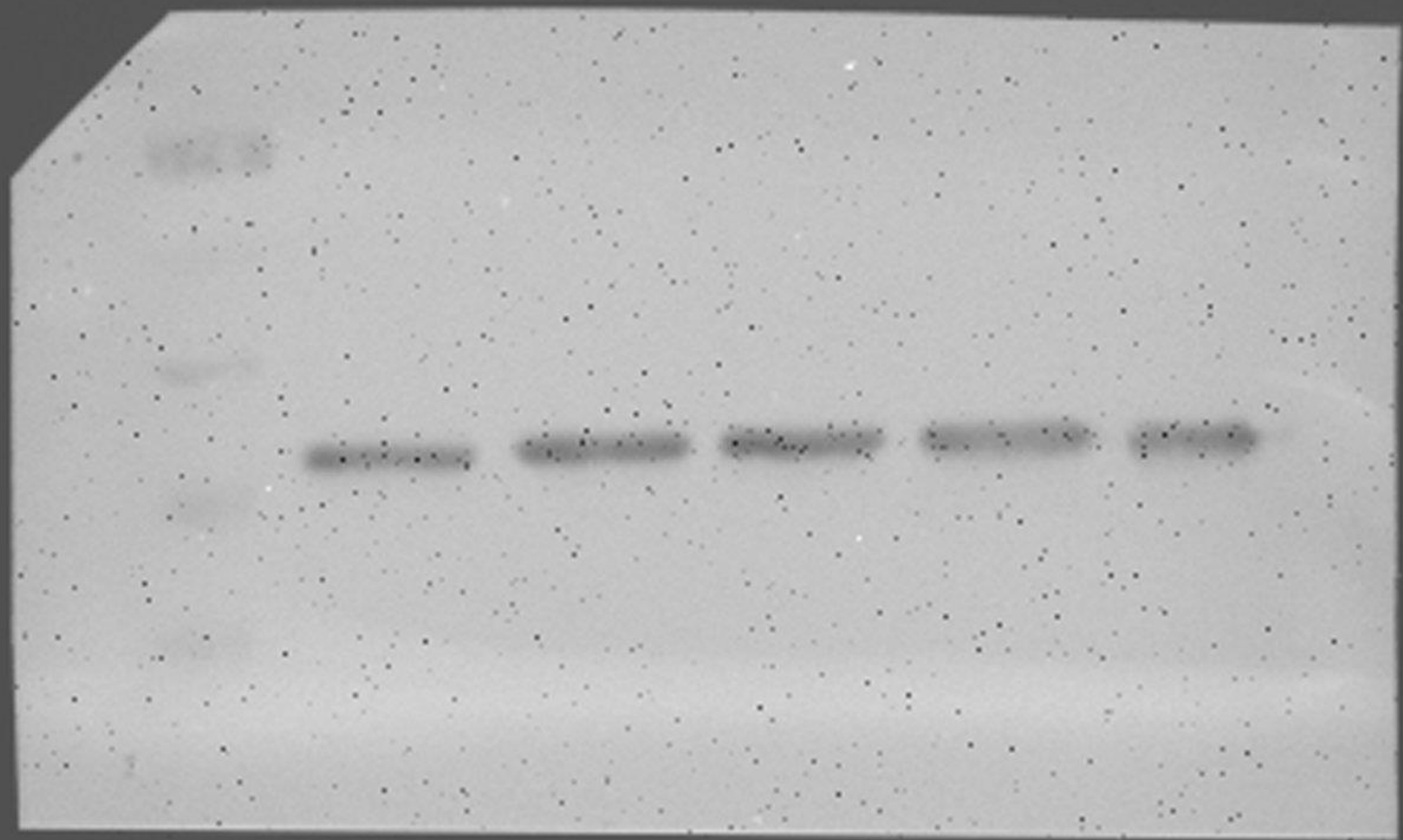

Supplement: Figure 3—source data 2. [file elife-95229-fig3-data2.zip › Figure 3-source data 2. Raw unedited gels for Figure 3/Figure 3-source data 4. Raw unedited gels for Figure 3.pdf]

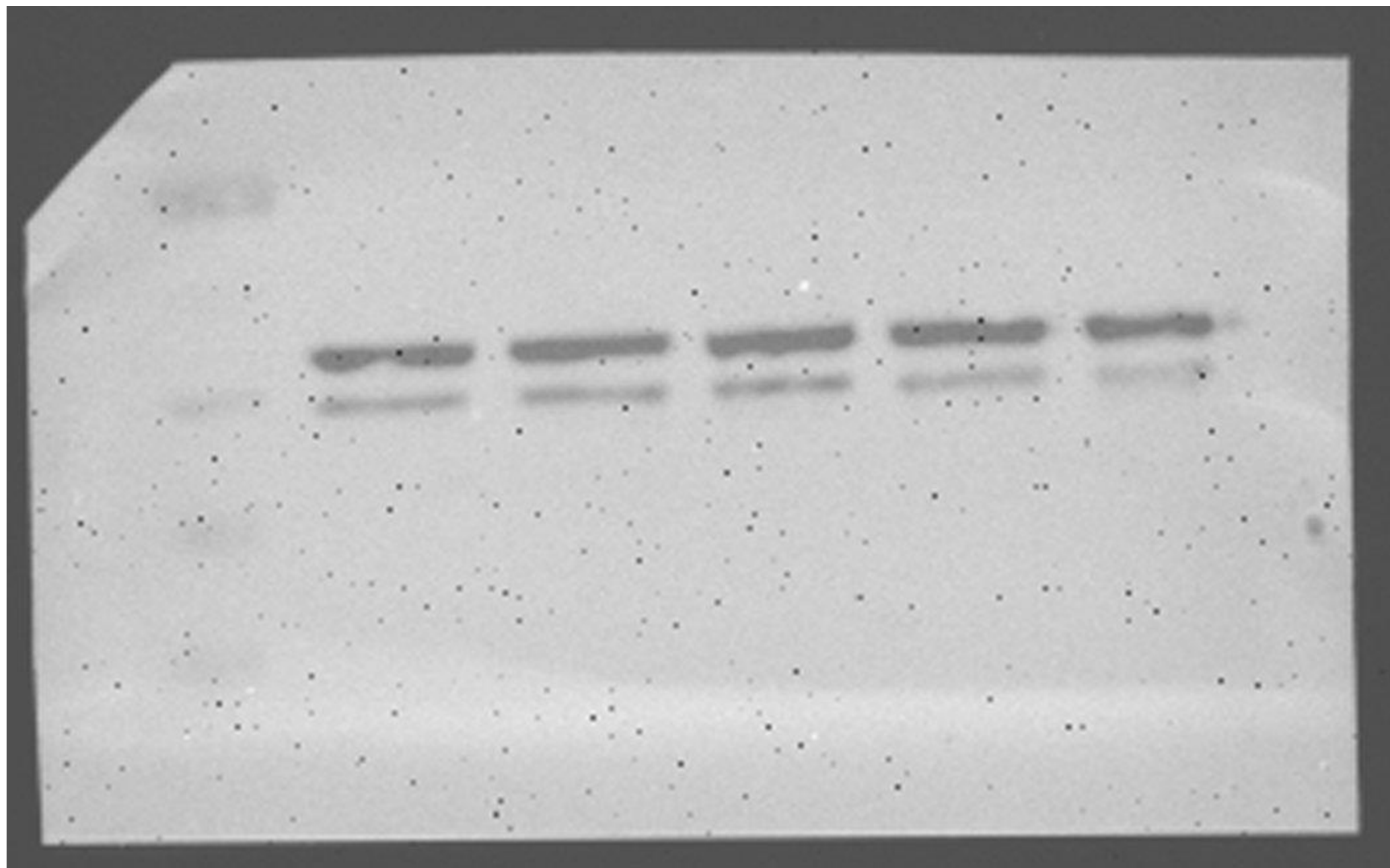

Supplement: Figure 3—source data 2. [file elife-95229-fig3-data2.zip › Figure 3-source data 2. Raw unedited gels for Figure 3/Figure 3-source data 5. Raw unedited gels for Figure 3.pdf]

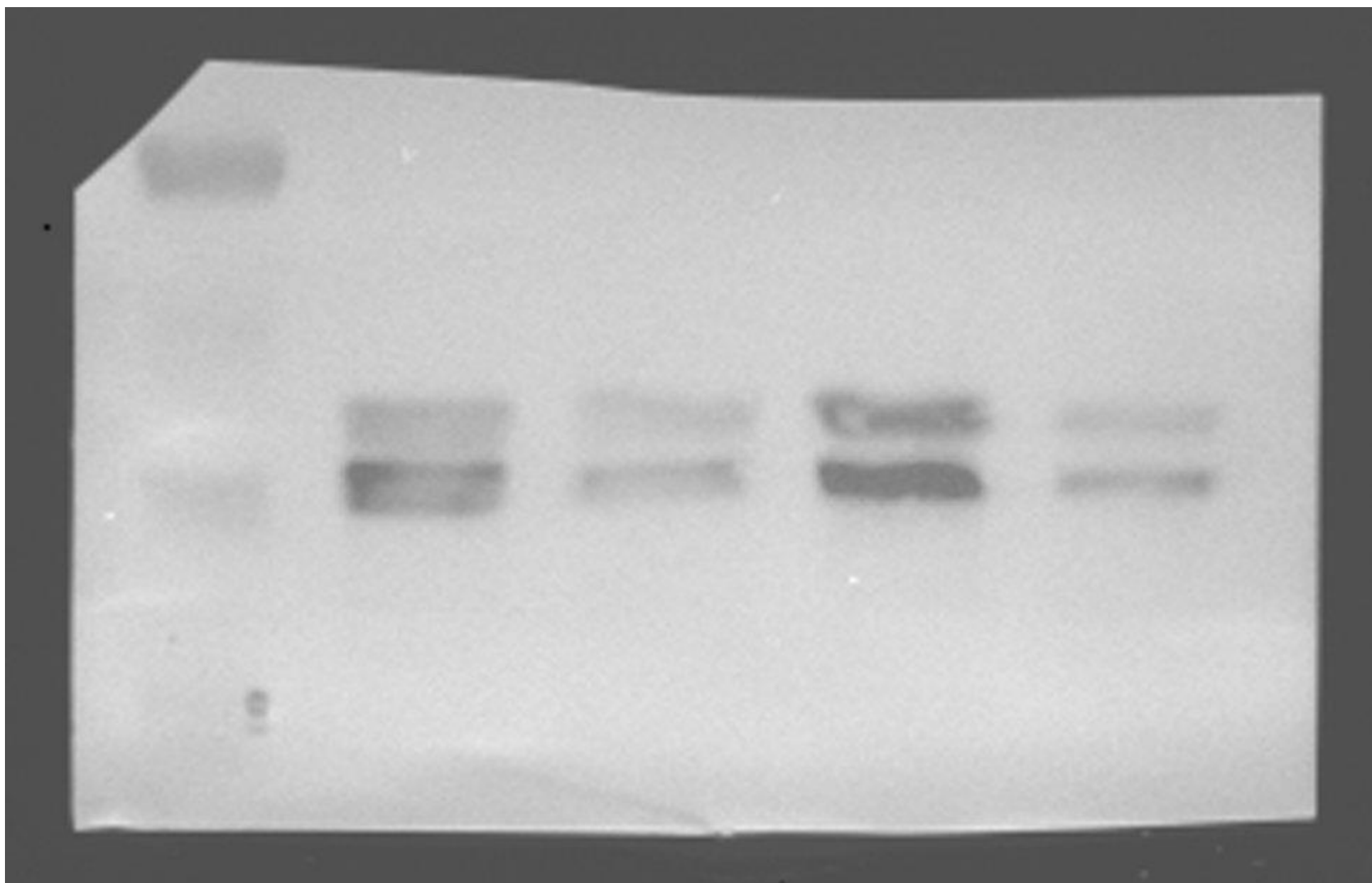

Supplement: Figure 3—source data 2. [file elife-95229-fig3-data2.zip › Figure 3-source data 2. Raw unedited gels for Figure 3/Figure 3-source data 6. Raw unedited gels for Figure 3.pdf]

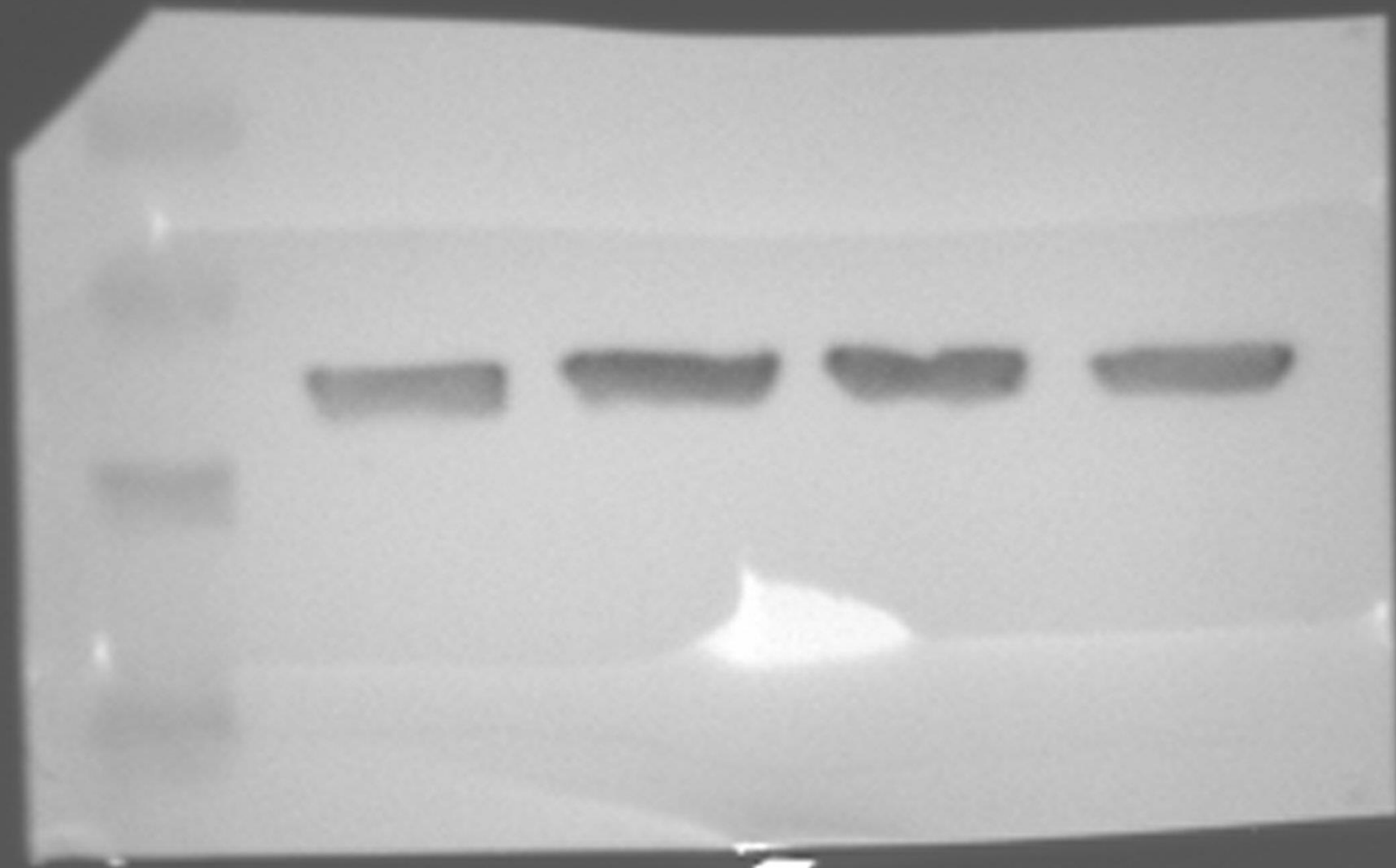

Supplement: Figure 3—source data 2. [file elife-95229-fig3-data2.zip › Figure 3-source data 2. Raw unedited gels for Figure 3/Figure 3-source data 7. Raw unedited gels for Figure 3.pdf]

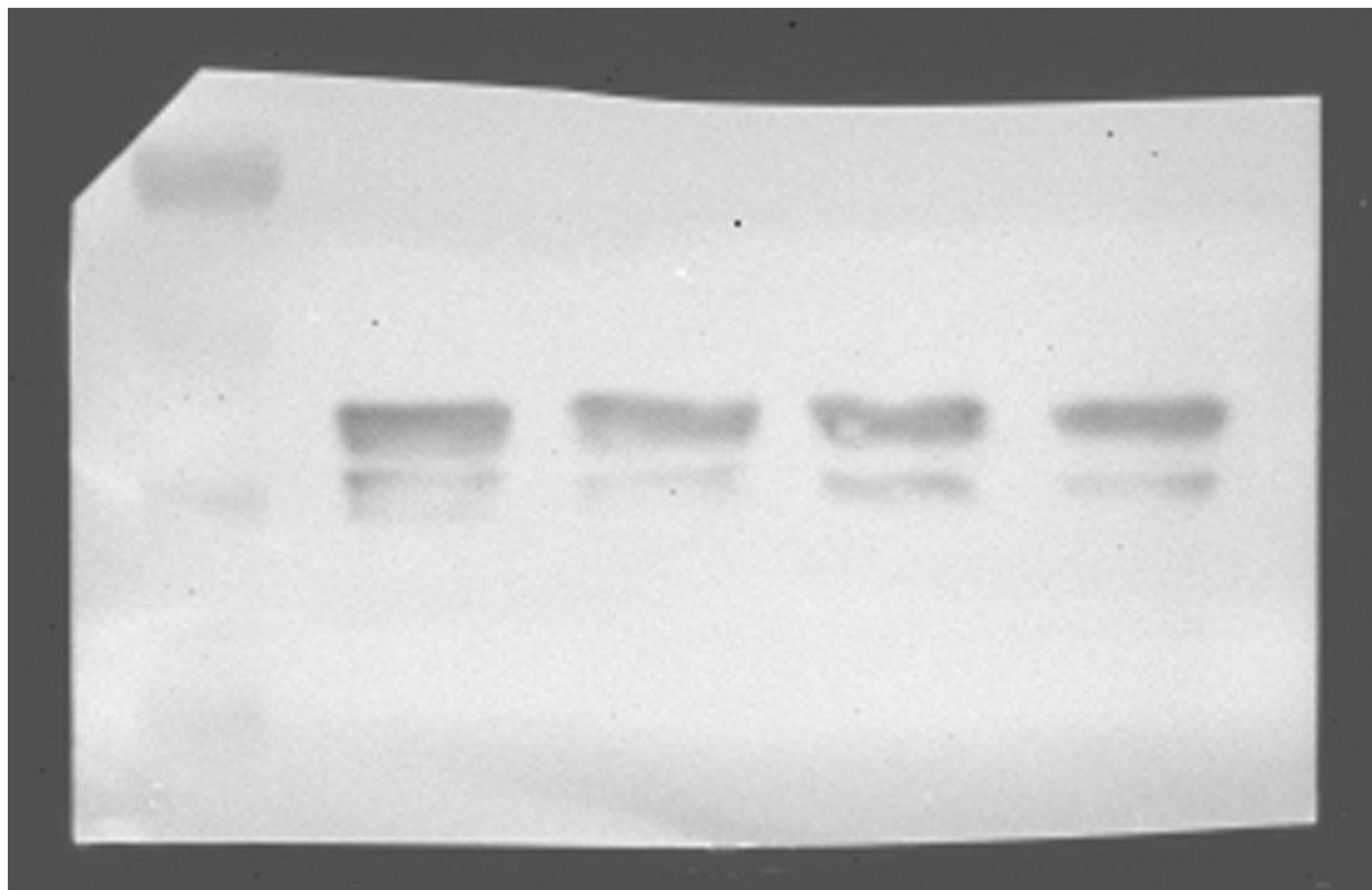

Supplement: Figure 3—source data 2. [file elife-95229-fig3-data2.zip › Figure 3-source data 2. Raw unedited gels for Figure 3/Figure 3-source data 8. Raw unedited gels for Figure 3.pdf]

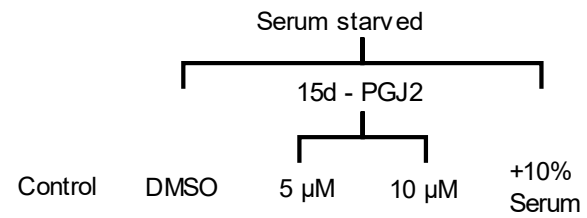

70 kDa  
50 kDa  
42 kDa

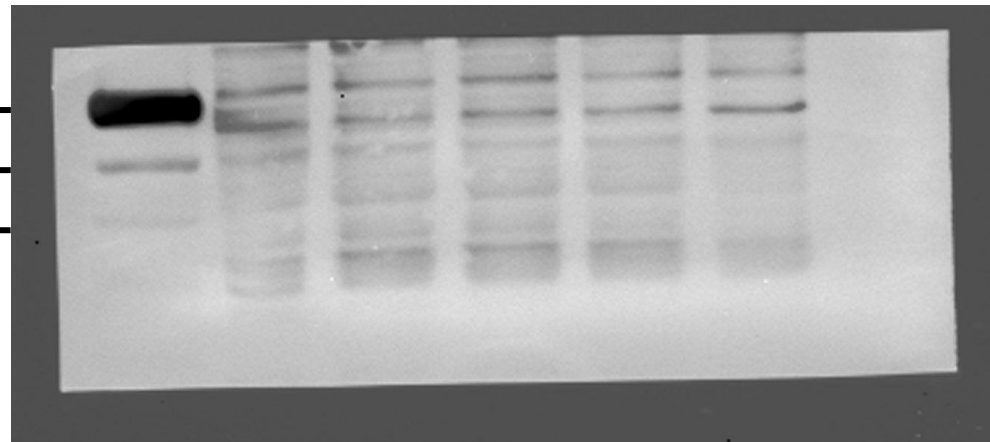

phosphoAkt

70 kDa  
50 kDa  
42 kDa

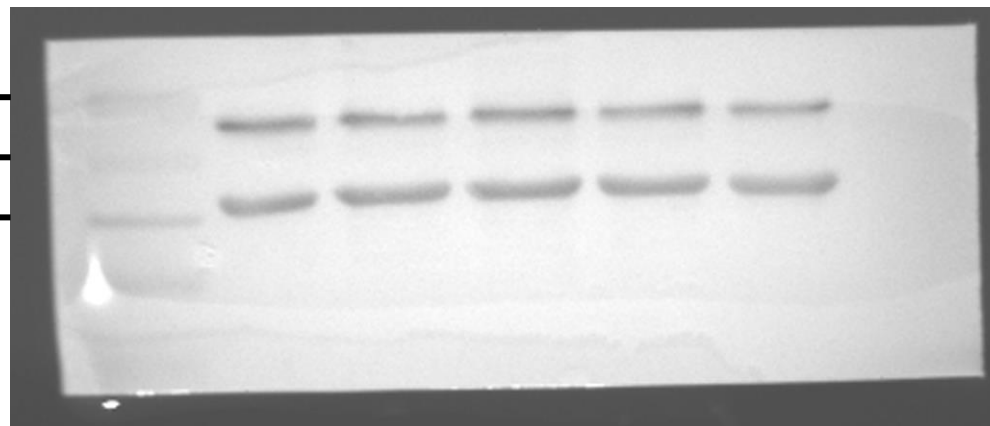

Akt  
 $\beta$ -Actin

Supplement: Figure 3—figure supplement 1—source data 1. [file elife-95229-fig3-figsupp1-data1.zip › Figure 3-figure supplement 1-source data 1. Uncropped and labelled gels for figure 3-figure supplement 3/Figure 3-figure supplement 1-source data 1. Uncropped and labelled gels for figure 3-figure supplement 3.pdf]

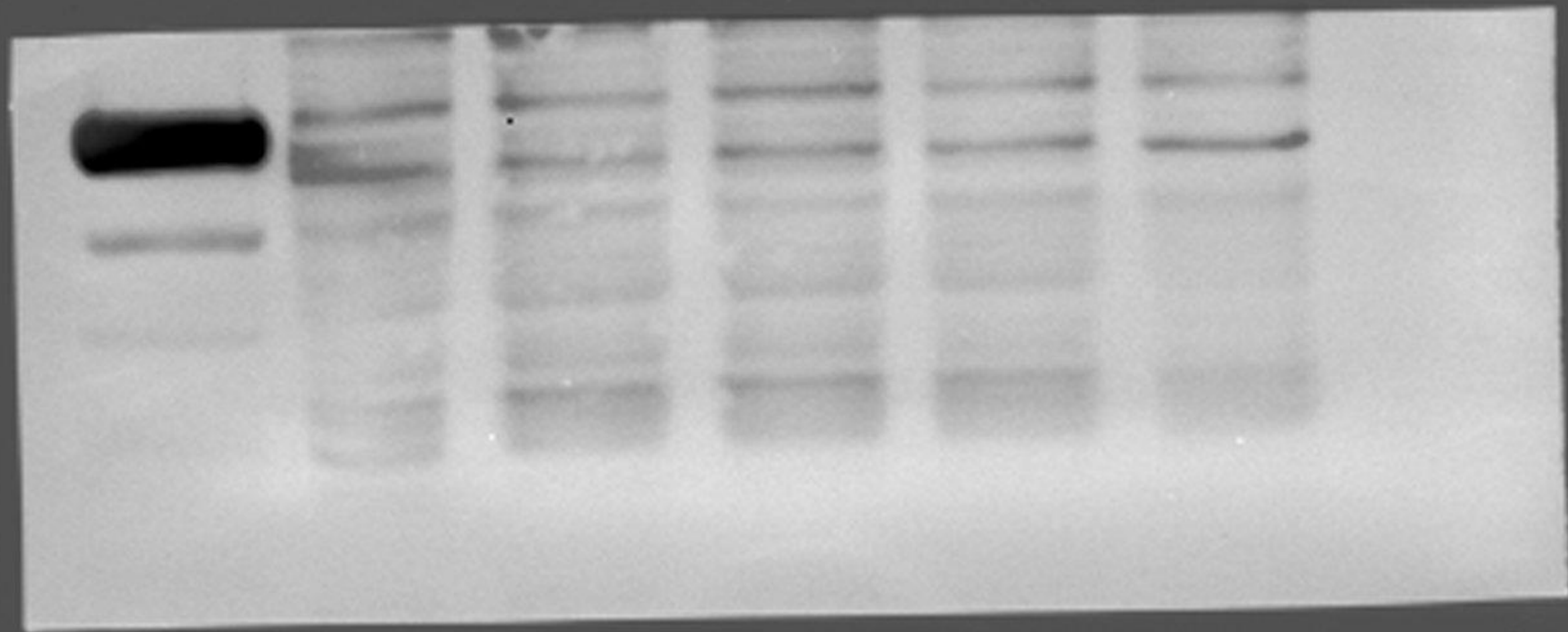

Supplement: Figure 3—figure supplement 1—source data 2. [file elife-95229-fig3-figsupp1-data2.zip › Figure 3-figure supplement 1-source data 2. Raw unedited gels for figure 3-figure supplement 1/Figure 3-figure supplement 1-source data 1. Raw unedited gels for figure 3-figure supplement 1.pdf]

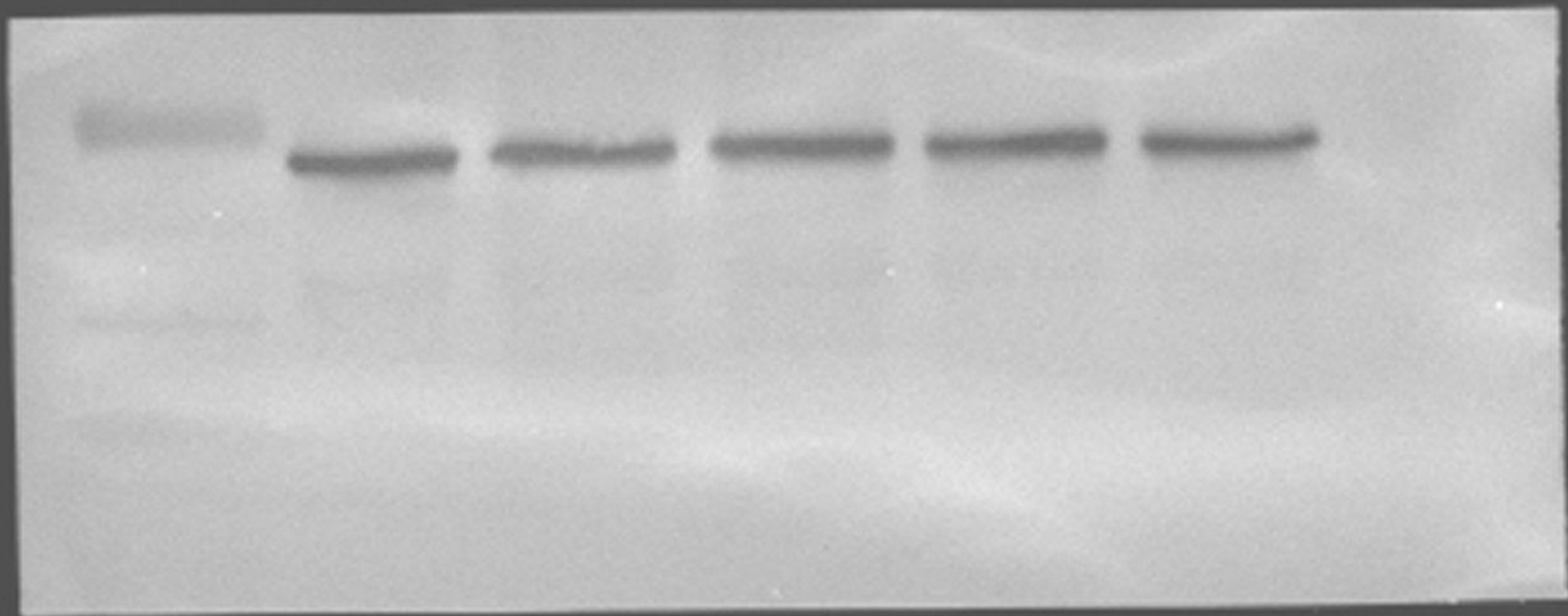

Supplement: Figure 3—figure supplement 1—source data 2. [file elife-95229-fig3-figsupp1-data2.zip › Figure 3-figure supplement 1-source data 2. Raw unedited gels for figure 3-figure supplement 1/Figure 3-figure supplement 1-source data 2. Raw unedited gels for figure 3-figure supplement 1.pdf]

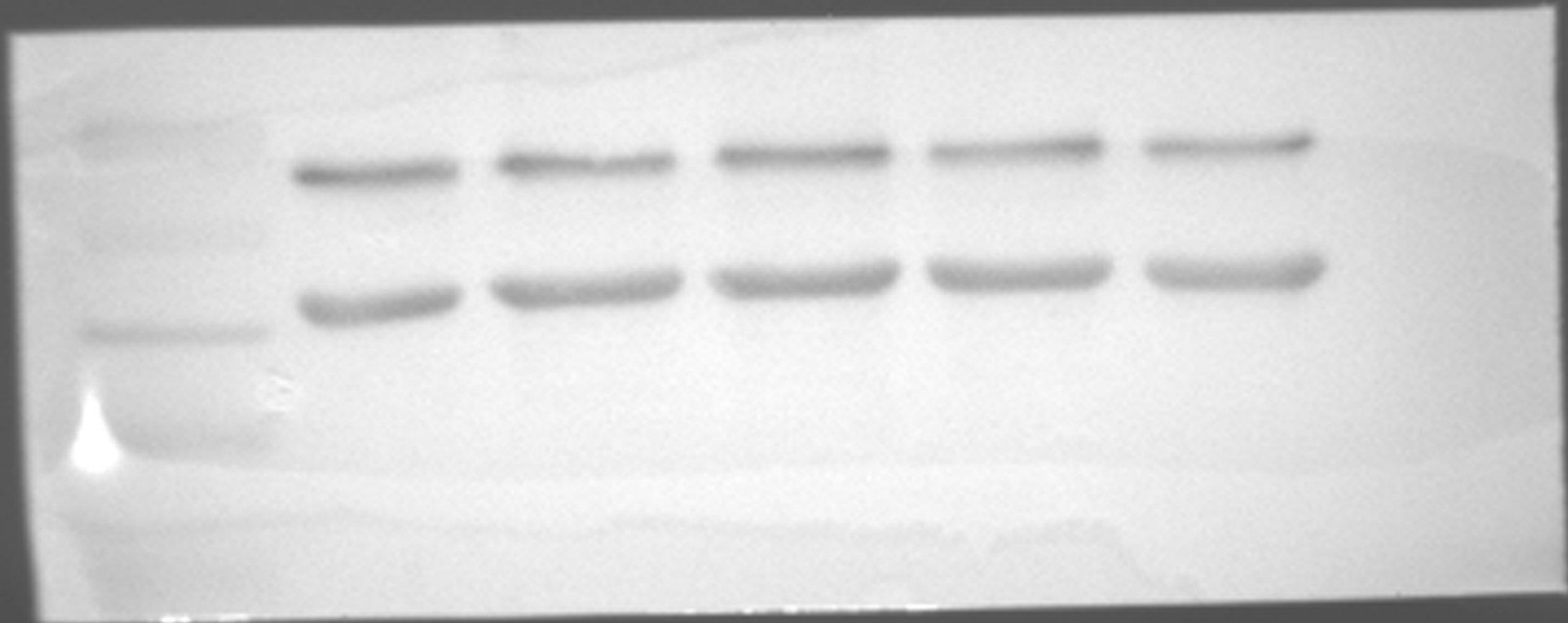

Supplement: Figure 3—figure supplement 1—source data 2. [file elife-95229-fig3-figsupp1-data2.zip › Figure 3-figure supplement 1-source data 2. Raw unedited gels for figure 3-figure supplement 1/Figure 3-figure supplement 1-source data 3. Raw unedited gels for figure 3-figure supplement 1.pdf]

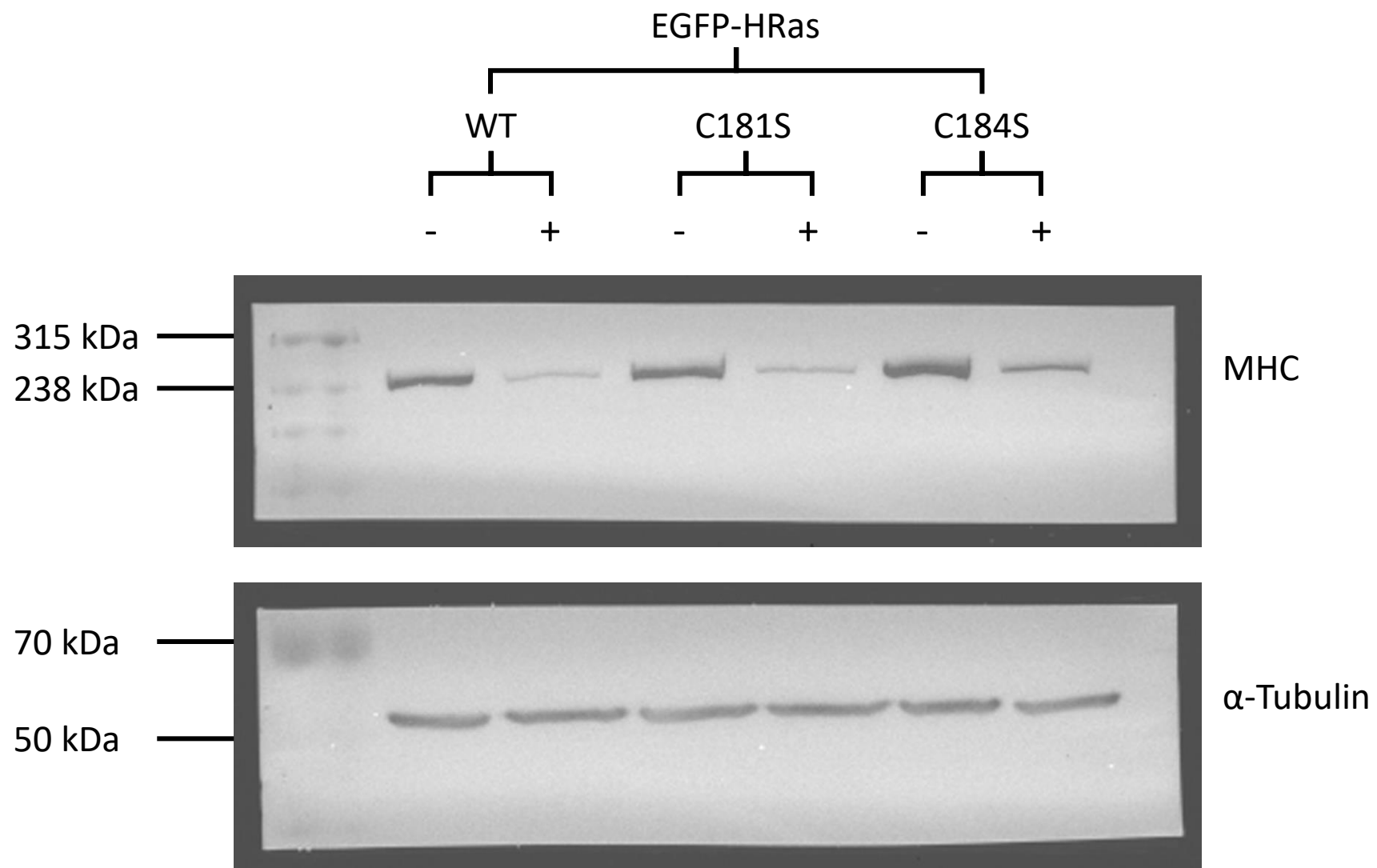

Supplement: Figure 4—source data 1. [file elife-95229-fig4-data1.zip › Figure 4-source data 1. Uncropped and labelled gels for Figure 4/Figure 4-source data 1. Uncropped and labelled gels for Figure 4.pdf]

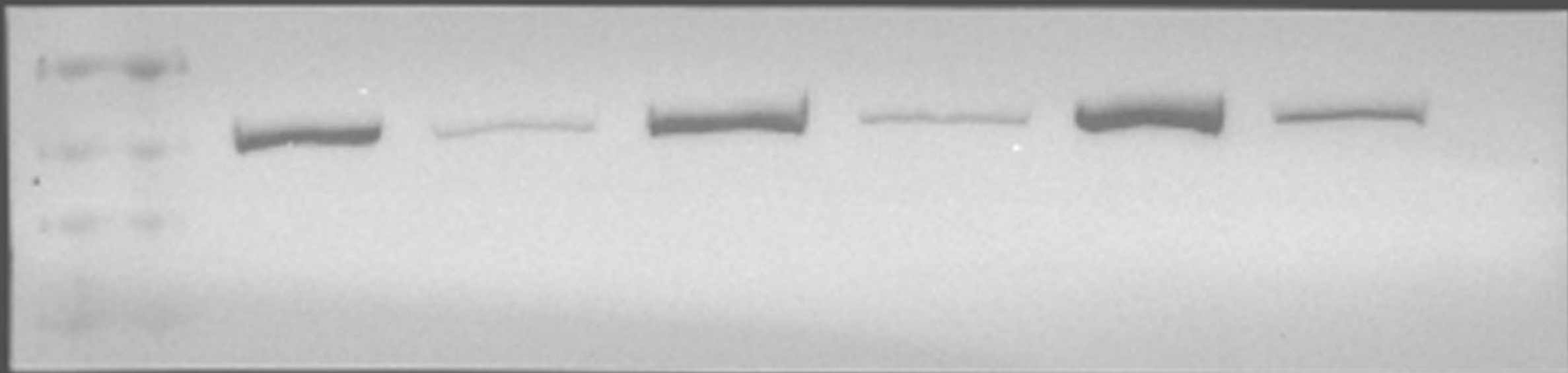

Supplement: Figure 4—source data 2. [file elife-95229-fig4-data2.zip › Figure 4-source data 2. Raw unedited gels for figure 4/Figure 4-source data 1. Raw unedited gels for figure 4.pdf]

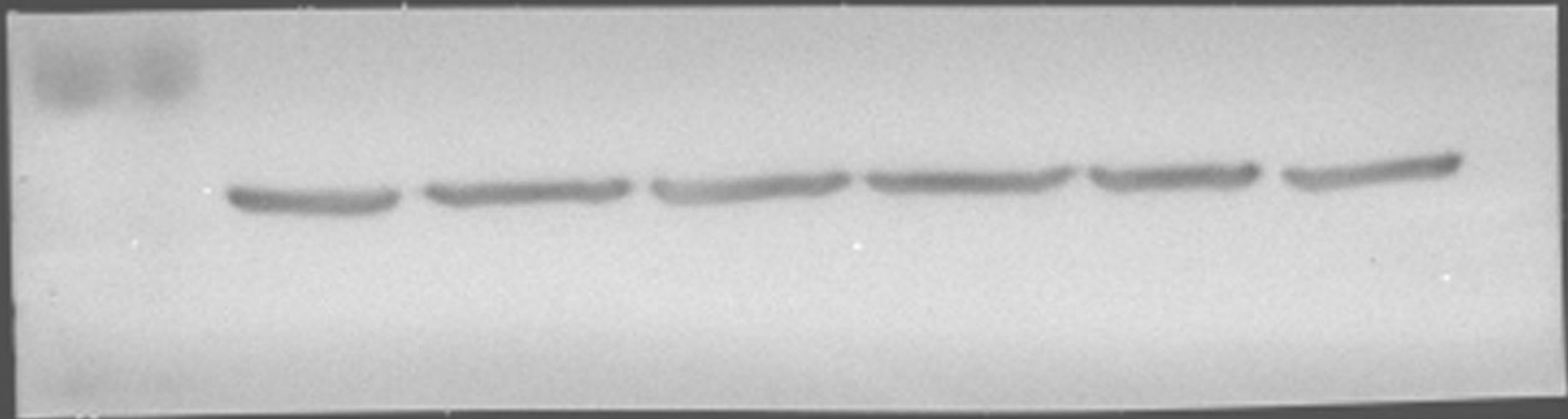

Supplement: Figure 4—source data 2. [file elife-95229-fig4-data2.zip › Figure 4-source data 2. Raw unedited gels for figure 4/Figure 4-source data 2. Raw unedited gels for figure 4.pdf]

Biological Replicate

| 1         |       |       |  | 2         |       |       |  | 3         |       |       |  |
|-----------|-------|-------|--|-----------|-------|-------|--|-----------|-------|-------|--|
| EGFP-HRas |       |       |  | EGFP-HRas |       |       |  | EGFP-HRas |       |       |  |
| V12       | V12   | V12   |  | V12       | V12   | V12   |  | V12       | V12   | V12   |  |
|           | C181S | C184S |  |           | C181S | C184S |  |           | C181S | C184S |  |

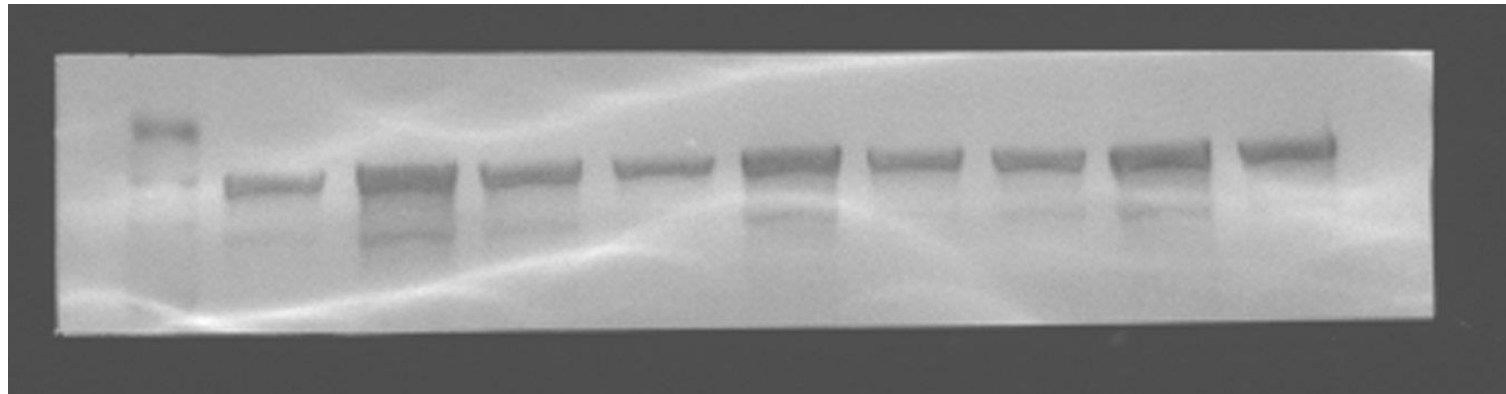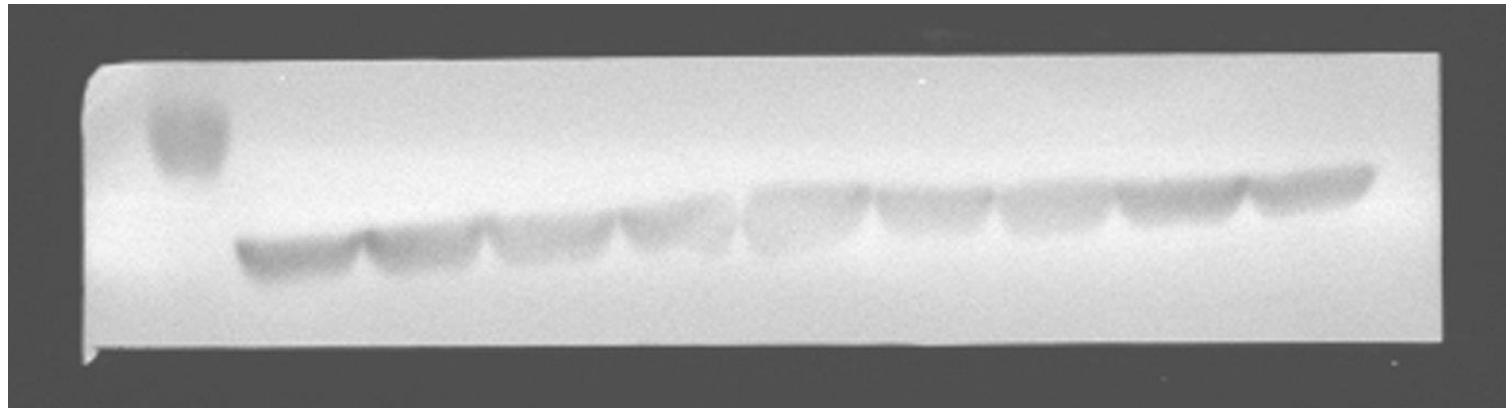

Supplement: Figure 4—figure supplement 1—source data 1. [file elife-95229-fig4-figsupp1-data1.zip › Figure 4-figure supplement 1-source data 1. Uncropped and labelled gels for figure 4-figure supplement 1/Figure 4-figure supplement 1-source data 1. Uncropped and labelled gels for figure 4-figure supplement 1.pdf]

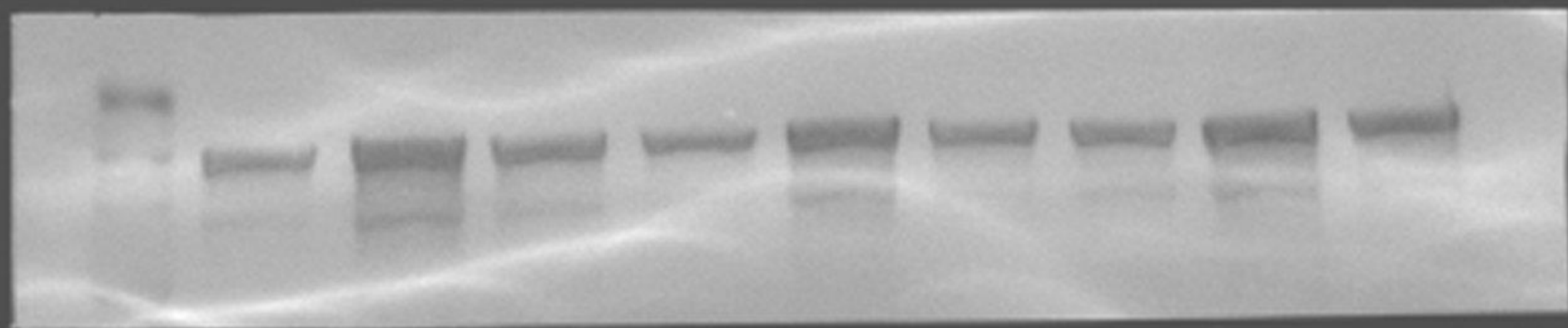

Supplement: Figure 4—figure supplement 1—source data 2. [file elife-95229-fig4-figsupp1-data2.zip › Figure 4-figure supplement 1-source data 2. Raw unedited gels for figure 4-figure supplement 1/Figure 4-figure supplement 1-source data 1. Raw unedited gels for figure 4-figure supplement 1.pdf]

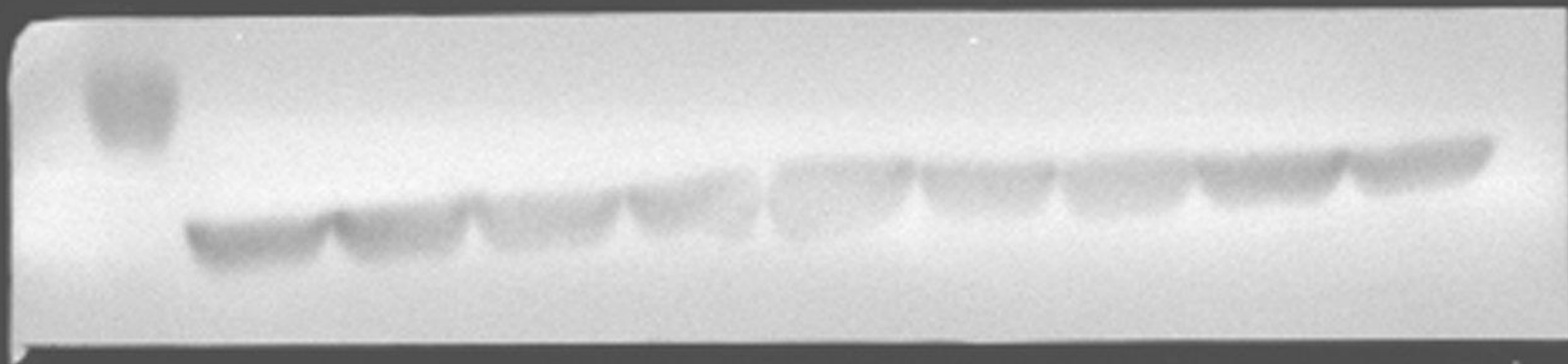

Supplement: Figure 4—figure supplement 1—source data 2. [file elife-95229-fig4-figsupp1-data2.zip › Figure 4-figure supplement 1-source data 2. Raw unedited gels for figure 4-figure supplement 1/Figure 4-figure supplement 1-source data 2. Raw unedited gels for figure 4-figure supplement 1.pdf]
