# Supplementary material for: Senescent cells inhibit mouse myoblast differentiation via the SASP-lipid 15d-PGJ2 mediated modification and control of HRas
Source: eLife. 2024 Aug 28;13:RP95229. doi: 10.7554/eLife.95229 (PMC11357351; doi:10.7554/eLife.95229)
Supplement: Figure 3—source data 1. [file elife-95229-fig3-data1.zip › Figure 3-source data 1. Uncropped and labelled gels for Figure 3/Figure 3-source data 2. Uncropped and labelled gels for Figure 3.pdf]

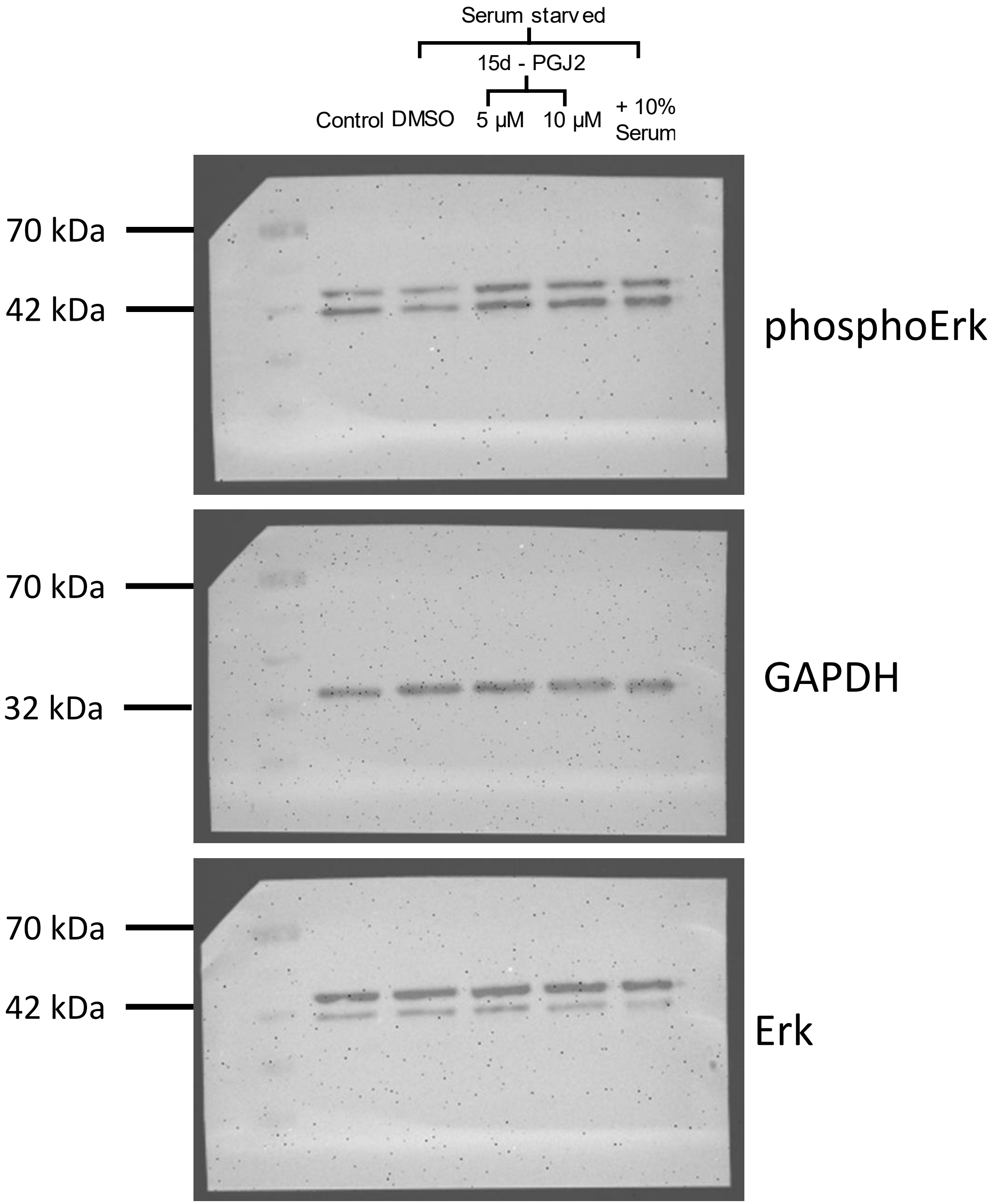

Serum starv ed
15d - PGJ2
Control
DMSO
5 µM
10 µM
+ 10% Serum
70 kDa
42 kDa
phosphoErk
70 kDa
32 kDa
GAPDH
70 kDa
42 kDa
Erk
