# Supplementary material for: Senescent cells inhibit mouse myoblast differentiation via the SASP-lipid 15d-PGJ2 mediated modification and control of HRas
Source: eLife. 2024 Aug 28;13:RP95229. doi: 10.7554/eLife.95229 (PMC11357351; doi:10.7554/eLife.95229)
Supplement: Supplementary file 1. — (A) List of primers used for qPCR. (B) List of reagents and the catalog numbers. (C) List of antibodies used for the western blot, immunoprecipitation, and immunofluorescence. [file elife-95229-supp1.docx]

**Supplementary file 1A: List of Primers.**

| **Primer Name** | **Primer Sequence (5’ – 3’)** |
| --- | --- |
| HRas WT Fwd | CCGCTCGAGCTATGACGGAATATAAGCTGG |
| HRas WT Rev | CCGGAATTCTCAGGAGAGCACACACTTGC |
| HRas C181S Rev | CCGGAATTCTCAGGAGAGCACACACTTGCAGCTCATGCTGCCGG |
| HRas C184S Rev | CCGGAATTCTCAGGAGAGCACACACTTGCTGCTCATG |
| mm Rn18s qPCR Fwd | CCCGTTGAACCCCATTCGTG |
| mm Rn18s qPCR Rev | GGGCCTCACTAAACCATCCA |
| mm Myod1 qPCR Fwd | TCCGCTACATCGAAGGTCTG |
| mm Myod1 qPCR Rev | GTCCAGGTGCGTAGAAGGC |
| mm Myog qPCR Fwd | CGATCTCCGCTACAGAGGC |
| mm Myog qPCR Rev | GTTGGGACCGAACTCCAGT |
| mm Myhc qPCR Fwd | TAAACGCAAGTGCCATTCCTG |
| mm Myhc qPCR Rev | GGGTCCGGGTAATAAGCTGG |
| mm Ptgs1 qPCR Fwd | TTACTATCCGTGCCAGAACCA |
| mm Ptgs1 qPCR Rev | CCCGTGCGAGTACAATCACA |
| mm Ptgs2 qPCR Fwd | TTCCAATCCATGTCAAAACCGT |
| mm Ptgs2 qPCR Rev | AGTCCGGGTACAGTCACACTT |
| mm Ptgds qPCR Fwd | GAGTACGCTCTGCTATTCAGC |
| mm Ptgds qPCR Rev | GGTTGGGGCAGGAAAACAATG |
| mm Cdkn2a qPCR Fwd | GCTCAACTACGGTGCAGATTC |
| mm Cdkn2a qPCR Rev | GCACGATGTCTTGATGTCCC |
| mm Cdkn1a qPCR Fwd | CCTGGTGATGTCCGACCTG |
| mm Cdkn1a qPCR Rev | CCATGAGCGCATCGCAATC |
| mm Cxcl1 qPCR Fwd | ACTGCACCCAAACCGAAGTC |
| mm Cxcl1 qPCR Rev | TGGGGACACCTTTTAGCATCTT |
| mm Cxcl2 qPCR Fwd | CCAACCACCAGGCTACAGG |
| mm Cxcl2 qPCR Rev | GCGTCACACTCAAGCTCTG |
| mm Tnfa qPCR Fwd | CCTGTAGCCCACGTCGTAG |
| mm Tnfa qPCR Rev | GGGAGTAGACAAGGTACAACCC |
| mm Il6 qPCR Fwd | CTGCAAGAGACTTCCATCCAG |
| mm Il6 qPCR Rev | AGTGGTATAGACAGGTCTGTTGG |
| mm Tgfb1 qPCR Fwd | CTTCAATACGTCAGACATTCGGG |
| mm Tgfb1 qPCR Rev | GTAACGCCAGGAATTGTTGCTA |

**Supplementary file 1B: List of Reagents**

| **Item Description** | **Manufacturer** | **Catalog No.** |
| --- | --- | --- |
| pEGFP-C1 | Clontech |  |
| Phusion high fidelity DNA polymerase | Thermo Scientific | F 530 |
| Xho1 restriction enzyme | New England Biolabs Inc. | R0146 |
| EcoR1 restriction enzyme | New England Biolabs Inc. | R0101 |
| T4 DNA Ligase | Takara Bio | 2011 |
| DMEM High Glucose | Gibco | 11995065 |
| FBS, Certified (Origin: United States) | Gibco | 16000044 |
| Horse Serum, Heat Inactivated (Origin: New Zealand) | Gibco | 26050070 |
| Penicillin – Streptomycin – Glutamine (100x) | Gibco | 10378016 |
| Penicillin – Streptomycin (100x) | Gibco | 15140163 |
| DPBS, no calcium, no magnesium | Gibco | 14190144 |
| 0.25% Trypsin - EDTA | Gibco | 25200056 |
| jetPRIME transfection reagent | Polyplus | 101000046 |
| Doxorubicin | Sigma – Aldrich | D1515 |
| 15-deoxy-Δ^12,14^-Prostaglandin J_2_ | Cayman Chemical Company | 18570 |
| 9,10-dihydro-15-deoxy-Δ^12,14^-Prostaglandin J_2_ | Cayman Chemical Company | 18590 |
| 15-deoxy-Δ^12,14^-Prostaglandin J_2_-Biotin | Cayman Chemical Company | 10141 |
| Dynabeads^TM^ MyOne^TM^ Streptavidin C1 | Invitrogen | 65001 |
| cOmplete Protease inhibitor cocktail tablets | Roche | 11697498001 |
| Wheat germ agglutinin, Alexa fluor 633 conjugate | Invitrogen | 2307289 |
| ProLong Gold Antifade Mounting medium | Invitrogen | P36930 |
| Paraformaldehyde | Sigma – Aldrich | 158127 |
| TRIzol reagent | Invitrogen | 15596018 |
| PrimeScript™ 1st strand cDNA Synthesis Kit | Takara Bio | 6110A |
| PowerUp™ SYBR™ Green Master Mix | Applied Biosystems | A25742 |
| WesternBrightTM ECL-spray Western blotting detection system | advansta | K-12049-D50 |
| Fetuin (Bovine) | Sigma – Aldrich | F2379 |
| hEGF | Sigma – Aldrich | E9644 |
| N2 Supplement | Thermo Scientific | 17502048 |
| Dexamethasone | Sigma – Aldrich | D4902 |
| DMEM Low Glucose | Thermo Scientific | 10567014 |
| Bovine Serum Albumin Fraction-V, Cell culture tested | HIMEDIA | 9048468 |
| Pierce^TM^ Streptavidin Magnetic Beads | Thermo Scientific | 88816 |

**Supplementary file 1C: List of Antibodies**

| **Antibody Name** | **Clonality** | **Species of Origin** | **Manufacturer** | **Catalog No.** |
| --- | --- | --- | --- | --- |
| Phospho-Erk (Thr202/ Tyr204) | Polyclonal | Rabbit | Cell Signaling Technology | 9101S |
| Erk | Polyclonal | Rabbit | Cell Signaling Technology | 9102S |
| GAPDH | Monoclonal | Mouse | Puregene | PG23002 |
| β-actin | Polyclonal | Rabbit | Cell Signaling Technology | 4967S |
| Phospho-Akt (Ser473) | Monoclonal | Mouse | Cell Signaling Technology | 4051S |
| Akt | Monoclonal | Rabbit | Cell Signaling Technology | 4691S |
| GFP | Monoclonal | Rabbit | Cell Signaling Technology | 2956S |
| Myosin Heavy Chain | Monoclonal | Mouse | Invitrogen | 14-6503-82 |
| Myosin Heavy Chain | Monoclonal | Mouse | Developmental Studies Hybridoma Bank (DSHB) | MF20 |
| p21 | Monoclonal | Mouse | Santacruz Biotechnology | sc-6246 |
| γH2A.X | Polyclonal | Rabbit | Novus Biologicals | NB100384 |
| Tubulin | Monoclonal | Mouse | Cell Signaling Technology | 3873S |
| HRP - Anti-Mouse |  | Horse | Cell Signaling Technology | 7076S |
| HRP - Anti-Rabbit |  | Goat | Cell Signaling Technology | 7074P2 |
| Alexa Fluor 568 – Anti-Mouse | Polyclonal | Goat | Invitrogen | A11031 |
